# DNA Methylation in Lung Cancer: Mechanisms and Associations with Histological Subtypes, Molecular Alterations, and Major Epidemiological Factors

**DOI:** 10.3390/cancers14040961

**Published:** 2022-02-15

**Authors:** Phuc H. Hoang, Maria Teresa Landi

**Affiliations:** Division of Cancer Epidemiology and Genetics, National Cancer Institute, NIH, DHHS, Bethesda, MD 20892, USA

**Keywords:** lung cancer, DNA methylation, epigenetics, hypermethylation, hypomethylation, tobacco smoking

## Abstract

**Simple Summary:**

Aberrant DNA methylation is associated with multiple malignancies, including lung cancer. Differences in methylation patterns have been observed across lung cancer subtypes, the mutational status of cancer driver genes, and various epidemiological factors. This review summarizes current knowledge of DNA methylation related to lung cancer, providing a foundation for improving prevention, diagnosis, and treatment strategies for this lethal disease.

**Abstract:**

Lung cancer is the major leading cause of cancer-related mortality worldwide. Multiple epigenetic factors—in particular, DNA methylation—have been associated with the development of lung cancer. In this review, we summarize the current knowledge on DNA methylation alterations in lung tumorigenesis, as well as their associations with different histological subtypes, common cancer driver gene mutations (e.g., *KRAS*, *EGFR*, and *TP53*), and major epidemiological risk factors (e.g., sex, smoking status, race/ethnicity). Understanding the mechanisms of DNA methylation regulation and their associations with various risk factors can provide further insights into carcinogenesis, and create future avenues for prevention and personalized treatments. In addition, we also highlight outstanding questions regarding DNA methylation in lung cancer to be elucidated in future studies

## 1. Introduction

**Figure 1:**
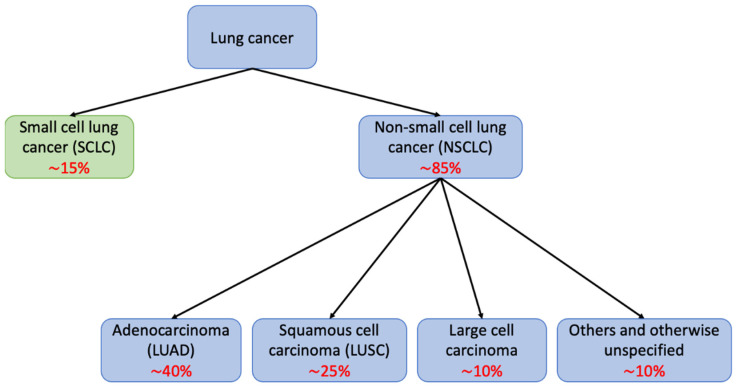
**Schematic diagram showing major histological classifications in lung cancer.** Lung cancer can be divided into small cell lung cancer (SCLC) and non-small cell lung cancer (NSCLC), which can be further classified into adenocarcinoma, squamous cell carcinoma, large cell carcinoma, and others. In red, approximate frequency of each subtype.

Lung cancer is the second most common malignancy and the leading cause of cancer-related mortality worldwide, with more than 2.2 million newly diagnosed cases and 1.9 million deaths in 2020 [1]. Most lung cancers can be classified into one of two main histological groups: non-small cell lung cancer (NSCLC) and small cell lung cancer (SCLC); these account for approximately 85% and 15% of all lung cancer cases, respectively (Figure 1). NSCLCs are further divided into different histological subtypes, with lung adenocarcinoma (LUAD) being the most prevalent (40%), followed by lung squamous cell carcinoma (LUSC) (25%), and large cell carcinoma with or without neuroendocrine features (10%) [2]. Despite recent advances in early detection and treatment, most patients are diagnosed at advanced stages with a significantly worse prognosis than in early stages [3], and the 5-year survival rate remains poor (22% for all stages combined) [4].

Multiple genetic and epigenetic alterations have been attributed to the development and progression of different lung cancer subtypes. SCLC is often characterized with concomitant inactivation of both tumor suppressor genes (TSGs) *TP53* and *RB* [5]. While *TP53* and *CDKN2A* are frequently mutated in LUSC, LUAD is commonly disrupted with *KRAS*; *EGFR*; TSGs *TP53*, *KEAP1*, *STK11*, and *NF1* mutations [6]. Epigenetic changes, including DNA methylation, histone modification, non-coding RNA expression, and DNA methylation have also been reported in lung cancer. For instance, compared to normal lungs, tumors exhibit H4K5/H4K8 hyperacetylation, H4K12/H4K16 hypoacetylation, and a loss of H4K20me3 [7]. MicroRNAs miR-196a and miR-200b, together with long non-coding RNAs *MALAT1* and *HOTAIR,* have been reported to be overexpressed in lung cancer [8]. Among the epigenetic factors, DNA methylation (DNAm) is the most well-studied epigenetic mechanism which could regulate gene expression through altering chromatin structure and transcription factor (TF) binding. DNA methylation could be detected by various methods, and several key factors (e.g., the study aim; amount and quality of DNA available; sensitivity and specificity requirements; cost) should be considered to choose the most appropriate one for a study (reviewed in detail elsewhere [9]). Changes in the DNAm levels of various genes have been observed in lung cancer across different histological subtypes [10,11,12,13], mutational status of common driver genes [14], smoking history [12,15], sex [16], and race/ethnicity [17,18]. In addition, DNAm of single candidate genes or sets of genes, as well as epigenetic subtypes, might be linked to prognosis [19,20,21]. DNAm states, therefore, emerged as potential therapeutic targets and powerful biomarkers for early detection, prognosis, and treatment responses in lung cancers (reviewed in detail elsewhere [22]). For instance, *SHOX2* and *RASSF1A* double gene methylation displayed much higher specificity (>90%) and sensitivity (>70%) for early lung cancer detection than traditional cytological method [23,24]. Other studies also suggest that methylation of sets of dual genes *RASSF1A* and *RARβ2*; *SHOX2* and *PTGER4*; or *p16* and *RARβ2* are feasible biomarkers for early diagnosis [25,26,27,28]. Certain methylation markers specifically associated with lung cancer progression and metastasis, such as increased methylation of *DAL-1, EPHB6*, *HS3ST2*, *TMEM88* and *MGMT*, and decreased methylation of *ELMO3,* are linked to higher rates of metastasis in NSCLC [29,30,31,32,33,34].

Treatment options for lung cancer patients, including surgery, chemotherapy, radiotherapy, immunotherapy, and targeted therapy, are determined by various factors such as tumor histology, stage, and molecular features [35]. While surgery is the main treatment for early stages of NSCLC, most patients are diagnosed with advanced lung cancer and cannot undergo curative resection. These patients have historically been given platinum-based chemotherapy and/or radiation as a standard first-line of treatment. Despite promising improvement in patients’ survival, only a subset of patients could benefit from immunotherapy (e.g., those with higher tumor mutational burden (TMB), high PD-L1 expression, and microsatellite instability) [36]. Some studies have shown that DNAm markers could potentially provide guidance for choosing additional therapeutic options, or help with selecting patients who could benefit from specific treatments. For instance, methylation levels of *APC*, *HOXA9*, *RARβ2* and *RASSF1A* could assist in better defining lung cancer subtypes (SCLC vs. NSCLC) and stage to develop corresponding treatment plans [37]. DNAm was also found to be highly correlated with TMB in NSCLC, suggesting that DNAm could be a potential alternative/complementary biomarker for immunotherapies. Additionally, DNAm markers also enable drug efficacy prediction. While high *IGFBP-3* methylation suggests cisplatin-resistant cells [38], high *RASSF1A* methylation might indicate a better response towards gemcitabine in NSCLC [39]. Furthermore, the alkylating agent temozolomide could be more effective in SCLC with *MGMT* methylation [40].

In this review, we summarize major findings on DNAm alterations in lung cancers, focusing on their mechanisms in lung tumorigenesis and specific associations with different lung histological subtypes, mutational status, and major epidemiological factors. Finally, we will discuss major outstanding questions that remain to be investigated in the field.

## 2. DNA Methylation Dysregulation in Lung Cancer

**Figure 2:**
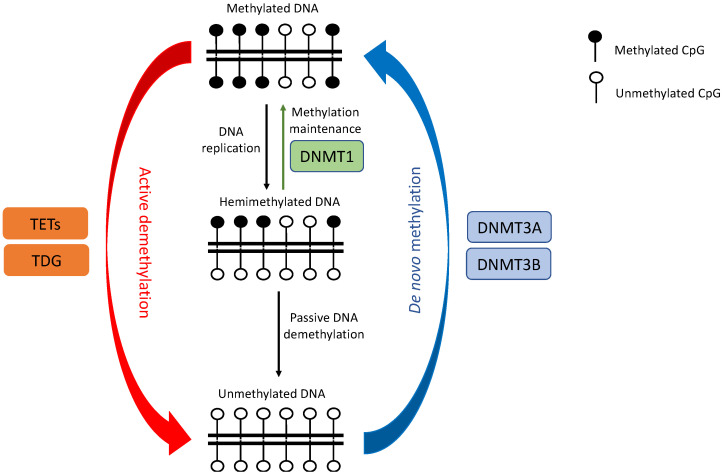
**Schematic drawing of DNA methylation dynamics by DNMTs and TETs, adapted from Jeltsch and Jurkowska [42].** DNA methylation patterns consisting of both methylated and unmethylated CpGs could be created de novo by DNMTs, typically DNMT3A and DNMT3B. The methylation pattern is maintained during DNA replication by DNMT1 but could be lost through passive or active DNA methylation, which is facilitated by TETs and TDG enzymes. TETs: ten–eleven translocation enzymes; DNMT: DNA methyltransferase; TDG: thymine–DNA glycosylase.

**Figure 3:**
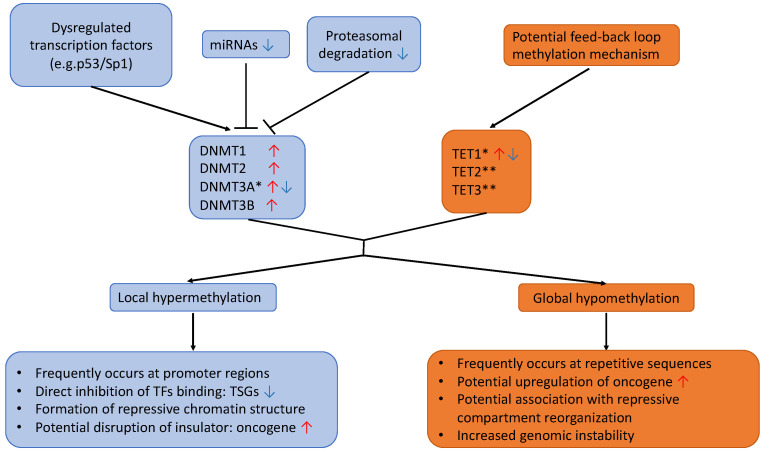
**Possible mechanisms and consequences of DNA methylation alterations in lung cancer.** Multiple mechanisms have been attributed to altered expression of DNMTs in lung cancer, but less is known for TETs, with one model proposing that TET1 might be regulated through a feedback loop mechanism by promoter methylation. The resulting effects are frequent local hypermethylation at promoter regions and global hypomethylation, mostly at repetitive sequences. These steps could lead to multiple potential genomic consequences, including downregulation of TSGs, activation of oncogene, induction of large chromatin/compartment changes, and increased genomic instability. *: conflicting reports about expression in lung cancer in literature; **: TET2 and TET3 expression level in lung cancer are not well-described in the literature; DNMT: DNA methyltransferase; TET: Ten–eleven translocation enzyme; TSG: tumor-suppressor gene; TF: transcription factor. Red arrows indicate upregulation, while blue arrows indicate downregulation.

DNAm is characterized by the covalent addition of a methyl group to the carbon-5 position of a cytosine base (5mC) by DNA methyltransferase enzymes (DNMTs). In mammals, the modification preferentially occurs at genomic regions enriched with CpG dinucleotide contexts, called CpG islands (CGIs), frequently found close to or within gene promoter regions. Aberrant genome-wide DNA hypomethylation and local promoter-specific hypermethylation have been observed in most tumors, even at early stages of malignancy [41]. While a global decrease in CpG methylation is commonly believed to activate silenced oncogenes and retrotransposon elements, and increase genomic instability, TSGs are often inactivated by DNA hypermethylation. A recent stochastic model suggests that DNAm levels at each CpG site are dynamically regulated by the local activity of both DNMTs and DNA demethylases (e.g., TET enzymes) as well as DNA replication rate [42] (Figure 2). Dysregulation of DNMTs and TETs have both been linked to tumor transformation in lung cancers (Figure 3).

### 2.1. DNMTs Dysregulation

There are five known DNMTs in humans, each exhibiting different specificity towards methylated and unmethylated DNA: DNMT1, DNMT2, DNMT3A, DNMT3B, and DMNT3L. DNMT1 preferentially acts at hemimethylated CpG sites and is responsible for methylation maintenance on daughter DNA strands after DNA replication [43]. In contrast, DNMT3A and DNMT3B are involved in de novo DNAm during germ cell development and early embryonic stages. DNMT2 (or TRDMT1) does not methylate DNA, but multiple tRNAs [44]. DNMT3L lacks a catalytic domain, but can interact with DNMT3A and DNMT3B to enhance their methylation activity [45].

Overexpression of DNMTs has been frequently observed in lung cancer with upregulation of DNMT1, and is consistently and independently associated with poor prognosis [46,47,48]. Belinsky et al. demonstrated that reduction in DNMT1 decreases tobacco-carcinogen-induced lung cancer in a mouse model [49]. The depletion of DNMT1 and/or DNMT3B resulted in growth arrest, apoptosis, and reactivation of TSGs in lung cancer cell lines [50]. Despite DNMTs not being frequently altered somatically in lung cancers, multiple mechanisms are attributed to overexpression of DNMTs, including dysregulation of regulatory transcription factors (e.g., p53/Sp1) [48], downregulation of miRNAs (e.g., miR-101) that negatively regulate DNMTs [51], and impaired proteasomal degradation of DNMTs [52] (Figure 3). Interestingly, although DNMT3A has been reported to be overexpressed in lung cancers [47], there is evidence suggesting that DNMT3A might act as tumor suppressor. Husni et al. showed that a lack of DNMT3A facilitates tumor progression through demethylation of oncogenes, and higher expression indicates favorable prognosis in LUAD [53]. In concordance, the deletion of *DNMT3A* in a mouse model promotes LUAD tumor growth and progression [54]. It is unclear if the specific role and expression levels of DNMT3A depend on different lung cancer histological types, as one study observed that DNMT3A was upregulated in SCLC but not NSCLC cell lines [55]. The specific implications of DNMT2 and DNMT3L in lung cancer are lesser known, but DNMT2 was reported to have higher activation in SCLC compared to many other cancer types [56].

Mechanistically, there are different ways in which aberrant DNMT activity might contribute to lung carcinogenesis. As higher DNMT expression has been shown to positively correlate with cell proliferation, DNMT dysregulation is believed to disrupt the cell cycle. A study from Sato et al. suggests that overexpression of DNMTs reflects increased cell proliferation in human lung cancers [55]. DNMTs appear to be important for ribosome synthesis, which is required for proliferation through their involvement in maturation of rRNA [57] and maintaining the nucleolar compartment as the site for ribosome assembly [58]. In EGFR-mutated NSCLC, Wu et al. showed that DNMT1 promotes cell proliferation through methylating promoters, and thus, downregulating hMLH1 and hMSH2, which suppress the cell cycle [59]. 

In addition, TSGs could be silenced by DNAm through increased DNMT activity. This could occur by direct and/or indirect inhibition. Direct inhibition involves direct blockage of the interaction between methylated DNA and methylation-sensitive TFs. Alternatively, methylated DNA recruits the m5CpG-binding domain (MBD), which contain proteins to form a complex that blocks the binding of TFs to DNA [60]. Indirect inhibition involves the recruitment of HDAC to the MBD-CpG complex, resulting in the formation of repressive chromatin structures up to a few neighboring genes or a whole chromosome band [61,62].

Given the role of DNMTs in lung tumorigenesis, many DNMT inhibitors (DNMTis) have been developed and tested for lung and other cancers. Commonly used DNMTis, such as 5-Azacytidine (5-Aza-CR, azacytidine, AZA, Vidaza) and its derivative 5-Aza-2′-deoxycytidine (5-Aza-CdR, decitabine, DAC, Dacogen), are nucleoside analogs, and work by forming covalent bonds with DNMTs, inactivating these enzymes [57]. Promoter methylation is, therefore, not maintained after each cell replication cycle. Consequently, previously silenced genes, including TSGs, are expressed. With a high dose of DNMTis, the nucleoside analogs are incorporated into DNA and form bulky adducts, resulting in DNA replication-fork stalling, and eventually, cell death [63]. A Phase I trial of a combination of decitabine and the HDAC inhibitor valproic acid showed promising results in advanced NSCLC patients, but its toxicity warrants further investigation [64]. In addition, decitabine was shown to reverse gefitinib resistance through demethylation of *RASSF1A* and *GADD45β* promoters in NSCLC cell lines [65]. Antisense oligonucleotides hybridizing to 3′-untranslated regions and causing degradation of DNMT mRNAs have also been tested. MG98 lowered DNMT expression and induced growth arrest and apoptosis in lung cancer cell lines [50]. MiR-29s expression was associated with decreased DNMT3A and -3B expression, and increased expression of TSGs *FHIT* and *WWOX*, resulting in tumorigenicity inhibition in both *in vivo* and *in vitro* settings [66]. However, it should be noted that DNMT downregulation could result in increased genomic instability by induction of chromosomal translocations [67]. Therefore, careful consideration is required to balance the dual facets when targeting DNMTs.

### 2.2. TETs Dysregulation

TET (Ten–Eleven Translocation) enzymes catalyze the oxidation of 5mC, leading to the reversal of DNAm, which was initially thought to be an irreversible epigenetic event. The family of TET proteins consists of TET1, TET2, and TET3, which have the same catalytic activity but differ in their domain architectures [68]. Although driver mutations of TETs are infrequent in lung cancer, with TET2 having a higher frequency of somatic mutations compared to TET1 and TET3, an altered expression of TETs has been observed [68,69]. However, the expression changes in TETs, especially for TET2 and TET3, in lung cancer remain somewhat unclear, with conflicting observations for TET1 in cell lines and primary tumor models. Recent studies based primarily on cell line models proposed a feedback loop mechanism where TET1 is directly downregulated by a DNA promoter methylation mechanism when normal cells are exposed to carcinogens, further shifting the balance towards CpG methylation at TSGs (e.g., *ABE1*, *HOXA9*, *HOXA7*, *OGG1*, *TIMP*, and *XRCC1*) and their inactivation [70,71]. In contrast, a separate study reported that TET1 is frequently upregulated (from 2- to 90-fold in 40–70% tumors) and acts as an oncogene in LUAD and LUSC primary tumors with a p53 loss of function [72]. In addition, they found that TP53 with transversion mutations was unable to bind to the promoter and repress TET1. Therefore, the precise mechanisms of TETs’ activity in lung cancer remain to be elucidated; however, various factors, including mutational status and models used (cell lines vs. primary tumors), will need to be considered in future studies.

### 2.3. Hypomethylation

Like many other cancers, extensive global hypomethylation in lung tumors is seen specifically at repetitive sequences, including SINEs, LINEs, subtelomeric repeats, and segmental duplications [10,73]. Hypomethylation at LINE-1 was associated with worse prognosis and more advanced stages, but was independent of driver-gene mutations in LUAD [74,75]. Despite the fact that hypomethylation at transcriptional regulatory elements occurs less frequently than hypermethylation, hypomethylation in these regions might activate oncogenes. Demethylation of CpG sites within the CGIs of *SNCG* associated with upregulation was observed in multiple solid tumors, including lung cancer [76]. *SNCG* was later found to be involved in cancer cell migration and invasion [77]. An early study showed that *MAGE* genes were upregulated in 70–85% of NSCLC tumors and carcinogen-damaged lung epithelial cells, and such upregulation correlates with loss of methylation [78]. *MAGEs*’ overexpression is associated with poor prognosis and is required for tumor growth and metastases in lung cancer [79]. In addition to acting as oncogenes, *MAGEs* might also contribute to the initiation and maintenance of cancer stem cells [79].

Alternatively, hypomethylation in tumors might induce genomic instability by reactivating retrotransposon elements and/or chromosome rearrangements. A study in NSCLC showed that hypomethylation at the 3′ tandem repeat region of *HRAS* may contribute to gene loss [80]. Daskalos et al. demonstrated that increased hypomethylation of retrotransposable elements (LINE-1 and Alu) leads to their enhanced transcription and strongly correlates with increased genomic instability in NSCLC [81]. In concordance, increased global hypomethylation is also associated with higher mutation, copy number variation, and allelic imbalance burden, as well as Treg/CD8 ratio during lung cancer progression [82]. These results imply the potential role of DNA hypomethylation in increased chromosomal instability, mutagenesis, and altered microenvironment. In contrast, Johnstone et al. suggested that global hypomethylation is tightly linked with compact and relatively silent genome compartments [83]. The compartment reorganization resulted in the activation of anti-tumor immunity and the repression of genes involved in stemness, invasion, and metastasis across multiple cancers. Therefore, they proposed that the global hypomethylation observed in tumors might not be a consequence of malignancy, but of cumulative cell divisions, with tumor-suppressing rather than oncogenic roles.

### 2.4. Hypermethylation

While reports and mechanistic details on hypomethylation are limited, there is plethora of studies on the targets and roles of hypermethylation in lung cancers. The variations in results reported across different studies are due to the disparity in designs, materials (e.g., primary tumors vs. cell lines), heterogeneity (varied lung cancer histology types), and techniques (e.g., quantitative vs. qualitative approaches, different numbers of CpG loci, different array platforms, different statistical approaches) [84]. Nevertheless, various CGIs of potential TSGs have been consistently identified as hypermethylated in lung cancers [73,85,86,87]. These genes play crucial roles in cellular functions that are often dysregulated in cancer, including apoptosis (*CASP8*, *DAPK*, *TNFRSF6*, *DR4*, *DR5*) [88,89,90,91], cell cycle regulation (*CDKN2A/p16*, *PTEN*, *RASSF1A*) [91,92,93,94,95,96], DNA repair (*MGMT*, *MLH1*, *MSH2*) [97,98], regulation of signaling pathway (*APC*, *RARβ-2*, *RUNX3*, *SHOX2*) [96,99,100,101,102], and cell adhesion and invasion (*CDH1*, *CDH13*, *TSLC1*) [103,104] (Table 1). Many of these genes are re-activated following treatment with methylation inhibitors (e.g., 5-aza-2′ deoxycytidine) in lung cancer cell lines [105], further confirming their silencing by DNAm. Although methylation might not necessarily result in gene inactivation, many methylated targets have been proven useful as epigenetic markers in lung cancer [22]. 

Interestingly, there have been reports of both cancer-specific hyper- and hypomethylation in the same gene. For instance, the promoter of potential TSG *UNC5D* was hypermethylated while downstream introns were hypomethylated in lung cancer, suggesting a potential complex interplay of the two opposite mechanisms to regulate gene expression [73]. Furthermore, DNA hypermethylation does not always result in the inactivation of TSGs, but instead in the activation of tumor-promoting genes. TERT promoter hypermethylation was recently shown to upregulate TERT expression in 82% of tumor types, including lung cancers [106]. Although insulator disruption by hypermethylation leading to oncogene activation has been observed in gliomas and gastrointestinal tumors [107,108], the implications of such a mechanism remain to be explored in lung cancers.

While many of the reported genes are subjected to altered methylation in lung cancer in general, some of them are specifically associated with different lung cancer subtypes, smoking status, and molecular alterations. Understanding the specific associations of DNAm in individual lung cancer subgroups could help to elucidate distinct underlying mechanisms, provide useful biomarkers for diagnosis and prognosis, and guide personalized treatments.

**Table 1:** Selected list of genes with frequently altered methylation in lung cancer and their biological functions. NSCLC: non-small cell lung cancer; SCLC: small cell lung cancer.

Methylation Changes in Tumors	Gene	Pathways	Histological Type Reported	Reference
Hypermethylation	*APC*	Cell proliferation, migration, and cell adhesion	NSCL, SCLC	[109,110]
	*CASP8*	Apoptosis	SCLC	[90]
	*CDH1*	Cell adhesion	NSCL, SCLC	[111,112]
	*CDH13*	Cell adhesion	NSCLC, SCLC	[113]
	*CDKN2A/p16*	Cell cycle regulation	NSCLC, SCLC	[55,95]
	*DAPK*	Apoptosis	NSCLC	[92]
	*FHIT*	Cell proliferation and apoptosis	NSCLC, SCLC	[114,115]
	*GSTP1*	Detoxification	NSCLC, SCLC	[116,117]
	*MGMT*	DNA repair	NSCLC, SCLC	[116,118]
	*MLH1*	DNA repair	NSCLC	[98]
	*MSH2*	DNA repair	NSCLC	[98]
	*PTEN*	Cell cycle regulation	NSCLC	[119]
	*RARβ*	Cell differentiation and proliferation	NSCLC, SCLC	[99]
	*RASSF1A*	Cell cycle regulation, genomic-stability maintenance, apoptosis, cell migration and invasion	NSCLC, SCLC	[120,121]
	*RUNX3*	TGF-β/Wnt signaling pathway	NSCLC, SCLC	[100]
	*SEMA3B*	Cell adhesion	NSCLC, SCLC	[122,123]
	*SHOX2*	Cell differentiation and proliferation	NSCLC, SCLC	[102]
	*TERT*	Immortalization of cancer cells	Lung cancer	[106]
	*TGFBR2*	Signaling	NSCLC	[124]
	*TNFRSF6/Fas*	Apoptosis	SCLC	[90]
	*TRAIL-R1/DR4*	Apoptosis	SCLC	[90]
	*TSLC1*	Cell adhesion	NSCLC, SCLC	[125]
Hypomethylation	*MAGE*	Transcriptional regulation, cancer development and progression	NSCLC	[78]
	*SNCG*	Cell migration and invasion	Lung cancer	[76]

## 3. DNA Methylation in Different Histological Subtypes

### 3.1. Non-Small Cell and Small Cell Lung Cancer

With most lung cancer cases being NSCLC, many DNAm patterns described in lung cancer are dominated by those from this histological group. Despite accounting for only 10–15% of cases, SCLC is characterized by rapid proliferation and metastasis, and high lethality, suggesting the need for a better understanding of this subtype. To the best of our knowledge, there have not been any studies systematically comparing genome-wide DNAm profiles between NSCLC and SCLC. Comparing methylation results from independent studies could be challenging due to various technical and analytical variations. Early studies examining selected genes showed that the promoters of multiple genes are hypermethylated in both SCLC and NSCLC (Table 1) with different methylation frequencies. For instance, *APC*, *CDH13*, and *CDKN2A*/*P16* were more frequently hypermethylated in NSCLC than in SCLC [55,110,113]. In contrast, selected genes involved in apoptosis pathways (*CASP8*, *TNFRSF6/Fas*, and *TRAIL-R1/DR4*) were found to be methylated in SCLC cell lines and tumors, but not in NSCLC samples [90]. 

A global methylation study in primary SCLC tumors identified 73 gene targets enriched at neuroendocrine-specifying TF binding sites, including *NEUROD1*, *HAND1*, *ZNF423*, *REST*, and homeobox genes [85]. The authors, thus, hypothesized that DNAm could induce a differentiation defect of neuroendocrine cells and result in the transition of tumor progenitor cells toward SCLC. A subsequent study further demonstrated that the Polycomb group protein EZH2 is strikingly overexpressed in primary SCLC compared to other tumor types, and is strongly correlated with the overall promoter methylation [126]. EZH2 has been established to recruit DNMTs, highlighting the connection between EZH2 upregulation and aberrant DNAm in SCLC [127]. In addition, Sun et al. surveyed genome-wide DNAm in 18 NSCL tumor/normal pairs, and observed that 46.1% of hypermethylated differentially methylated regions (DMRs) overlap with poised promoters in embryonic stem cells (ESCs), including many homeobox and development-associated genes [128], as previously observed in another cohort [15]. The results suggest widespread methylation at homeobox- and development-associated genes, which is a shared mechanism of gene-methylation silencing facilitated by the Polycomb complex across lung cancer subtypes, including SCLC and NSCLC [86]. Although dysregulation of neuroendocrine-specifying TFs by methylation contributes to SCLC tumorigenesis, it is less clear whether, and how, aberrant methylation of cell-fate determination genes underly NSCLC development.

A correlation between methylation and gene expression could help to pinpoint cancer driver events in each subtype. DNAm was found to be one of the primary mechanisms responsible for upregulation of *BCL2* oncogene and downregulation of TSGs *RB1* and *TCF21* in SCLC [85,126]. Although there have been no suggestions of *BCL2* or *RB1* dysregulation by methylation in NSCLC, *TCF21* has been established to be silenced via hypermethylation in this subtype [128,129]. Early studies on NSCLC reported single gene or selected genes whose expressions are attributed to promoter hypermethylation (e.g., *CDKN2A*, *FHIT*, *DAPK*, and *RASSF1A*). A recent genome-wide study identified 20 potential hypermethylated driver genes (e.g., *SLIT2*, *CDO1*, *TCF21*, *PCHD17*, *IRX1*, *HSPB6*, and *TBX5*) and 13 methylation-driver non-coding RNAs in NSCLC [128]. Further studies characterizing and comparing DNAm driver events in SCLC and NSCLC tumors would be helpful for personalized treatments for each subtype.

Using DNAm markers for differential diagnosis is also a current active area of research. Walter et al. observed a significantly higher *RASSF1A* methylation level in SCLC and large cell neuroendocrine carcinomas than in NSCLC, suggesting it could be a potential marker for differential diagnosis [130]. In a recent study, Nunes et al. examined promoter methylation levels of four genes (*APC*, *HOXA9*, *RARβ2*, and *RASSF1A*) across lung cancer subtypes in circulating cell-free DNA (cfDNA) [37]. They found *HOXA9* and *RASSF1A* to have higher methylation levels in SCLC than NSCLC among 129 samples, displaying relatively high specificity and accuracy (>79%) but modest sensitivity (63.9% and 52.0%, respectively). With larger, independent cohorts and systematic exploration of useful markers from genome-wide methylation, DNAm analysis from circulating cfDNA could be proven useful in clinical settings. 

Interestingly, methylation patterns have also shown distinct associations with prognosis and treatment responses for different subtypes. For instance, hypermethylation at the *MGMT* promoter indicates that SCLC patients might benefit from treatment with the alkylating agent temozolomide, but patients with NSCLC would not [40]. The clinical utility of *RASSF1A* methylation has been shown in NSCLC but it is unclear in SCLC. NSCLC patients with methylated *RASSF1A* respond better to gemcitabine compared to patients with wild-type tumors [39]. In addition, patients with methylated *RASSF1A* have better survival when treated with paclitaxel compared to gemcitabine [131]. Unsupervised clustering of SCLC tumors led to the identification of a CGI methylator phenotype (CIMP) in 9/29 tumors with significantly poorer prognosis [132]. However, the association of a CIMP-high phenotype with worse clinical outcome is also analogous to that in NSCLC/adenocarcinoma [133]. 

### 3.2. Lung Adenocarcinoma and Squamous Cell Carcinoma

LUAD and LUSC are two histopathological subtypes that compose the majority of NSCLC. They are believed to have different cells of origins with distinct molecular characteristics, gene expression profiles [134], and therapeutic options [135]. Therefore, differences in DNAm patterns are expected and could potentially be exploited for better diagnosis and personalized treatments. Previous studies interrogating a list of selected genes suggest that methylation rates of *ANK1*, *APC*, *CCND2*, *CDH13*, *GATA3*, *KCNH5*, *LINE-1*, *RARβ*, *RASSF1*, and *RUNX3* are significantly higher in LUAD than in LUSC [13,17,136,137,138], while higher methylation frequencies of *AGTR1*, *CDKN2A/P16*, *DAPK*, *HOXA9, MLH1*, *SHOX2*, *SFMTB2*, *SFRP4*, *TIMP3*, *TGIF*, and *ZIC4*, are more often observed in LUSC compared to LUAD [13,94,98,102,139,140,141,142]. An early study using HumanMethylation27 BeadChips to examine 48 stage-I NSCLC tumors identified 263 hypermethylated CpG sites, and 513 hypomethylated CpG sites in LUSC compared to LUAD tumors [12]. However, the biological implications of the DNAm pattern differences were not discussed. Using TCGA data, Yang et al. found 391 genes with opposite methylation patterns in LUAD and LUSC tumors compared to normal tissues, with *CTSE* and *SLC5A7* also displaying opposite expression patterns [143]. These genes are generally enriched in nutrition metabolic pathways, leading the authors to suggest that the DNAm differences likely reflect differences in the LUAD and LUSC microenvironments. In contrast, a recent study using a cell-type deconvolution approach to identify differentially methylated cell types (DMCTs) observed that most cell-specific DNAm changes are largely independent of LUAD or LUSC subtypes [144]. Therefore, further investigations are required to establish whether there is substantial genome-wide difference in the DNAm landscape, and to clarify how it might contribute to the distinct characteristics of LUAD and LUSC.

Immunohistochemical staining of resected tumors is often used for differential diagnosis between LUAD and LUSC, but the sensitivity for even the most widely used marker, TTF-1, is only 62% [145]. In an effort to identify useful DNAm markers for differential diagnosis, Carvalho et al. found three DMRs significantly different between 20 LUAD and 27 LUSC tumors (*GAS1*, *FAM78B*, and *DOTL1*) [146]. However, the author concluded that testing of more DMRs in a larger dataset is required to construct a more accurate and distinguishing panel. A meta-analysis extracting methylation frequency from 151 studies on 108 genes suggests that two hypomethylated genes (*CDKN2A* and *MGMT*), and three hypermethylated genes (*CDH13*, *RUNX3*, and *APC*), when comparing LUAD to LUSC, might be able to distinguish the two subtypes [147]. Nevertheless, none of them have been independently validated as differential diagnostic biomarkers in large datasets. Shi et al. identified six genes (*CLDN1*, *TP63*, *TPX5*, *TCF21*, *ADHFE1*, and *HNF1B*) as potential DNAm markers for LUSC diagnosis compared to a non-tumor lung [148]. However, the authors did not evaluate whether these markers could distinguish between LUAD and LUSC. Using circulating cfDNA, Nunes et al. observed higher methylation of *HOXA9* promoter in LUSC than in LUAD, but the biomarker only displayed 55.2% sensitivity and 74.3% specificity for LUSC detection [37]. Therefore, robust DNAm markers to distinguish between the two subtypes remain to be identified and validated. Potentially, a combination of expression and methylation features might be proven useful to improve the classification [149]. 

In addition to the traditional histological classification, gene expression divides NSCLC to epithelial- and mesenchymal-like phenotypes with distinct responses to EGFR inhibitors (e.g., erlotinib) [150]. Walter et al. showed that genome-wide DNAm could further distinguish these expression-based phenotypes in both NSCLC cell lines and tumors [151], with the methylation status of *ERBB2* and *ZEB2* being especially predictive. 

There is limited information on the specific associations of DNA prognosis markers in LUAD and LUSC. In an exploratory analysis, Heller et al. observed that patients with methylated *HOXA2* and *HOXA10* LUSC have worse prognoses than those with wild-type LUSC [13]. In LUAD, gene expression upregulation associated with decreased methylation of *FAM83A* could indicate a poorer prognosis [152], while hypermethylation of *GRK6* could promote cell migration and invasion [153]. However, it is unclear if the same patterns would extend to LUSC tumors.

## 4. Smoking and DNA Methylation

It is well established that tobacco smoking is associated with higher risk for lung cancer (between 15- to 25-fold in comparison to never-smokers). Previous studies have suggested that smoking is highly related to SCLC development, with only 2.5% of SCLC cases occurring among non-smokers [154], in contrast to 15–25% in NSCLC [155]. There have been several studies demonstrating a strong link between smoking and lung tumorigenesis via DNAm. Gao et al. showed that smoking is associated with hypomethylation (*KLF6*, *TERT*, *MSH5*, *ACTA2*, *GATA3*, and *VTI1A*) and hypermethylation (*STK3A* and *CHRNA5*) of genes related to lung cancer susceptibility [156], suggesting they might be regulated by DNAm. Zhang et al. observed that hypomethylation at *AHRR* (cg05575921), 6p21.33 (cg06126421), and *F2RL3* (cg03636183) is associated with tobacco smoking and higher lung cancer risk [157]. Baglietto et al. identified three additional hypomethylated CpGs indicative of lung cancer risk (cg21566642 and cg05951221 in 2q37.1; and cg23387569 in 12q14.1) [158]. Interestingly, hypomethylation of most of these CpGs was lowest for current smokers, and increased with time after smoking cessation for former smokers [158].

Tobacco smoking has been shown to be associated with an increased DNAm of specific genes in somatic analyses of lung cancers (e.g., *p16*, *APC*, *MGMT*, *ANK1*, *MTHFR*) [17,94,137,159,160]. In contrast, Pulling et al. and Wu et al. showed that *MGMT* promoter methylation is more common in never-smokers than in smokers [161,162]. Although the reasons for the discrepancies are unclear, Liu et al. suggested the use of paraffin-embedded rather than fresh frozen tumors in Pulling et al. [159] could contribute to the differences. Alternatively, the MGMT methylation level might be associated with TP53-mutated tumors rather than with all lung tumors [162]. In addition, *RUNX3* was more frequently methylated in non-smokers [100]. Mechanistically, tobacco-smoke carcinogens are likely to increase DNMT1 expression through disrupting pathways involved in DNMT1 ubiquitination and degradation; this results in de novo promoter hypermethylation of TSGs and, eventually, tumorigenesis in lung cancers [163,164]. In addition, smoking might induce chronic inflammation and reactive oxygen species, leading to aberrant methylation followed by the recruitment of the silencing complex (DNMT1, DNMT3B, and members of Polycomb repressive complex 4) [165]. Furthermore, Vaz et al. demonstrated that chronic exposure to cigarette smoke induced abnormal methylation in human bronchial epithelial cells at genes enriched in stem cell differentiation and embryonic morphogenesis ontology [166]. They hypothesized that this exposure transitions cells to a more stem-cell-like nature via epigenetic changes, activating key signaling events and sensitizing cells to transformation by a single oncogenic event (e.g., *KRAS* mutations). 

There have been limited studies systematically comparing DNAm profiles in lung cancers from ever- and never-smokers, and most of the differences observed have been modest. Using a 30-gene panel, Pesek et al. observed *CDH13* being more methylated in non-smokers compared to smokers in advanced NSCLC [167]. An early genome-wide study using the Illumina Infinium HumanMethylation27 platform identified five genes as hypermethylated (*IRF8*, *IHH*, *LGALS4*, *IL18BP*, and *VTN*), but only *LGALS4* as downregulated in smokers compared to never-smokers LUAD [15]. Similarly, Bjaanæs et al. used Illumina 450K array and identified 225 CpGs from 147 unique genes as hypermethylated in ever-smokers in an independent LUAD cohort [14], but none of them displayed significant differential methylation in The Cancer Genome Atlas (TCGA) cohort. However, *LGALS4* was validated as hypermethylated and downregulated as seen previously [15]. Even though LGALS4 regulates cell–cell and cell–matrix interactions and was proposed to act as a tumor suppressor in colorectal cancer [168], its contribution in lung cancer is unclear. Using TCGA data, Bakulski et al. compared DNAm status in never-smokers with current and former LUAD smokers. For current smokers, they mapped multiple differentially methylated CpGs to small nucleolar RNA genes, ribosomal subunit genes, *MYO1G*, and *ZFP28* [169]. These CpGs were mapped to *MYO1G* and cytochrome p450 family genes (*CYP1A1*, *CYP1B1*) in former smokers. However, the author acknowledged that these results should be interpreted with caution given the modest sample size and cell type heterogeneity. Moreover, the differences might reflect epigenetic changes due to cancer progression rather than tobacco smoke exposure.

## 5. Molecular Status (*KRAS*, *EGFR*, *TP53* Mutations) and Methylation

*KRAS* mutations occur in 20–40% of NSCLC, with a higher prevalence in LUAD vs. LUSC (32% vs. 4%), Western vs. Asian (26% vs. 11%), smokers vs. non-smokers (30% vs. 10%), and almost never occurring in SCLC [170,171]. An early study showed an association between *KRAS* mutations and methylated *p16*, as well as a higher overall methylation index in LUAD [172]. In concordance, Selamat et al. showed that *KRAS* mutations were associated with an epigenetic subgroup in LUAD with higher methylation [15], reminiscent of a CIMP subgroup previously observed in colorectal cancer [173]. However, *KRAS* mutations did not appear to drive this subgroup, but more complex molecular mechanisms may be involved. In contrast, a genome-wide comparison between *KRAS*-mutated tumors and wild-type tumors only discovered two hypomethylated CpGs corresponding to *PFDN1* and *MAEA* genes [14]. Interestingly, a study showed that most LUAD and large cell carcinomas with *KRAS* mutations lack *RASSF1A* promoter methylation [174], but separate studies did not observe the same relationship [121,175]. In a recent study, induction of *KRAS* mutations in isogenic lung cancer cell lines resulted in stochastic DNAm changes in genes enriched in development and differentiation [176]. 

The frequency of *EGFR* mutation is much higher in NSCLC never-smokers compared to smokers (49.3% vs. 21.5%), females compared to males (43.7% vs. 24.0%), LUAD compared to non-LUAD (38.0% vs. 11.7%), and Asians compared to non-Asians (38.4% vs. 15–20%) [177]. With the increasing use of EGFR-TKIs in *EGFR*-mutated NSCLCs, a comprehensive understanding of the genetic and epigenetic profiles would be essential to fully exploit the treatment strategy. An early study showed that *EGFR*-mutated tumors had lower methylation indexes and were more often associated with unmethylated *p16* and *CDH13* [172]. Pesek et al. observed a greater methylation proportion of *APC*, *CDKN2B*, *ESR1*, and *VHL* in patients with *EGFR* mutations [167]. A study by Bjaanæs et al. identified 454 differentially methylated (mostly hypermethylated) CpGs within 275 unique genes in EGFR-mutant tumors compared to wild-type tumors. These genes are enriched in mTOR and EIF2 signaling pathways, which the authors suggest might explain the activation of the mTOR pathway and EGFR-TKIs in lung cancers with EGFR mutations. Using TCGA data, Xu et al. found that *EGFR* is significantly hypomethylated in LUAD tumors with *EGFR* mutations compared to those without [178]. In addition, nineteen hypomethylated CpGs and seven hypermethylated CpGs in EGFR-mutated groups, compared to wild-type, were also observed, but their biological implications were not discussed. They identified correlations between the methylation of *EGFR* and gene expression in both mutated and wild-type *EGFR* tumors, suggesting that *EGFR* could be epigenetically regulated. In fact, methylation at the EGFR promoter has been suggested to contribute to EGFR-TKI resistance [179].

The specific association between *TP53* mutations and DNAm in lung cancer is lesser known. Bjaanæs et al. identified 2026 hypomethylated and 349 hypermethylated CpGs corresponding to 834 unique genes in LUAD *TP53*-mutated tumors compared to wild-type tumors [14]. Although the underlying biological implications of these genes were not discussed in detail, a number of these genes were also identified as hypomethylated in *TP53*-mutated breast cancer such as *AFF3* [180]. *TP53* mutations were suggested to affect global DNAm through DNMT1 overexpression in lung cancer, and increased genomic instability, but how this is linked to the changes observed, largely of hypomethylation, is unclear. Interestingly, *TP53* mutations were also associated with hypomethylation in basal-like breast cancer [181], and CIMP in colorectal cancer [182].

## 6. Race/Ethnicity and Sex

Epidemiological studies have shown that lung cancer risks differ across ethnic/racial and gender groups, with African Americans and Native Hawaiians, and females vs. males, having the highest risks [183,184,185,186]. A study examining DNAm patterns from umbilical cord blood at birth in 201 newborns found that 13.7% of CpGs, highly represented in cancer pathways, display differential methylation between African Americans and Caucasians [187]. The authors suggest that some of the epigenetic precursors might exist at birth, partially explaining the differences in race-specific cancer risks. The differential association of race/ethnicity to DNAm in lung cancer have been examined in numerous studies. Toyooka et al. evaluated the methylation status of seven genes in 541 NSCLC and found higher methylation rates of *MGMT* and *GSTP1* for cases from the USA and Australia than in those from Japan and Taiwan [17] (Table 2). Another study pointed out that tobacco-smoking dose impacts blood DNAm at specific sites (e.g., *FOXK2*, *PBX1*, *FNDC7*, *FUBP3*) differentially between Native Hawaiian smokers vs. white/Japanese American smokers, which might explain some of the lung-cancer-risk variations [18]. Sun et al. found smoking-related genes *F2RL3* and *GPR15* to have similar methylation patterns in African American and Caucasian populations, suggesting common molecular mechanisms of epigenetic modification induced by environmental exposures across different populations [188]. 

A genome-wide analysis classified both LUAD and LUSC as cancers with strong sex-biased DNAm patterns, with differentially methylated genes enriched in various pathways, including immune response and apoptosis [16]. Although tobacco smoking was found to result in global hypomethylation in both sexes, the effect was stronger in females [189]. The gender-specific methylation of established genes frequently methylated in lung cancers have been reported (Table 2), but with some discrepancies. *RARβ* was found to have higher methylation frequency in females [138] and *RASSF1A* methylation levels in males with lung cancers [160], but this was not observed in a separate study [17]. While Wu et al. observed higher methylation of the *MGMT* promoter in males with NSCLC [162], consistent with Sarter et al. in a Singapore Chinese cohort [190], others have suggested otherwise [191]. A recent study suggested that a worse survival outcome in men might be influenced by the disruption of a sex-associated transcription network in a subset of NSCLC [192]. They hypothesized that the expression dysregulation is likely mediated by the widespread autosomal hypomethylation potentially caused by the deficiency of male-specific demethylase KDM5D, due to somatic chromosome Y loss.

**Table 2:** Selected aberrantly methylated genes in lung cancer with reported gender and or ethnicity differences.

Gene	Groups with Higher Methylated Frequency	Ethnic/Racial/Geographical Difference Reported	Sex Difference Reported
*CDH13*	Females		[167]
*ERα*	Males		[193]
*ESR1*	Females		[167]
*GATA5*	Females		[167]
*GSTP1*	USA/Australia higher than Japan/Taiwan	[14]	
*KCNH5*	Females		[138]
*KCNH8*	Females		[138]
*MGMT*	Conflicting reports, USA/Australia higher than Japan/Taiwan	[14]	[162]
*PAX6*	Females		[167]
*RARβ*	Conflicting reports		[17,138]
*RASSF1*	Conflicting reports		[17,160]

## 7. Conclusions and Perspectives

Undoubtedly, DNAm and its dysregulation play an essential role in lung cancer development. In this review, we have highlighted findings suggesting multiple underlying mechanisms resulting in aberrant DNAm in lung cancers. In addition, we have also described major specific associations of DNAm patterns across different lung cancer histological subtypes, and by tobacco smoking and driver-gene mutational status, race/ethnicity, and sex.

Despite recent advances in the field, several outstanding questions remain, which warrant investigation in future studies.

- The dysregulation of DNMTs, TETs, and other related proteins (e.g., Polycomb protein EZH2) play a major role in altering DNAm patterns in lung cancer, creating a window of opportunity for targeted drug development and treatment. However, as highlighted in our review, further studies are required to reconcile and elucidate the conflicting reports regarding their specific roles (e.g., DNMT3A, TETs). We suspect that the paradox might be attributed to various factors, including mutational driver status, model systems, and histological subtypes.
- In addition, there remains a knowledge gap in how different DNMT isoforms specifically contribute to lung cancer development. For instance, DNMTB has over 30 different isoforms with ∆DNMT3B4-del being the most abundant isoform in NSCLC [194]. In this study, the overexpression of ∆DNMT3B4-del led to increased global hypomethylation, local hypermethylation, and epithelial hyperplasia. However, the expression of ∆DNMT3B4-del alone was not sufficient to transform lung epithelial cells into tumor cells in animal models. In addition, the abnormal expression of other isoforms was also observed. Therefore, a better knowledge of the role of DNMT isoforms in lung cancer is important.
- The dynamic remodeling of DNAm is essential for lung development and cell fate decisions as stem cells exit pluripotency [195]. Many studies have shown that DNA hypermethylation at key developmental genes occupied by the Polycomb complex in embryonic stem cells is a common hallmark in many tumor types, including lung cancers [128,166,196]. The current model suggests that DNA hypermethylation could result in shifting the balance towards the silencing of these developmental genes. These genes are maintained at low expression in embryonic and adult stem cells; hence, their silencing contributes to a stem-like state with upregulated oncogenic pathways (e.g., KRAS/MAPK signaling) and to sensitizing cells to malignant transformation. The dysregulation of various lineage TFs, either through DNA methylation or somatic mutations, in combination with cancer-driver-gene mutations could potentially influence the formation of different lung cancer subtypes [197]. It has been shown that the dysregulation of neuroendocrine-specifying TFs by DNAm contributes to SCLC tumorigenesis [85]. It is important to fully comprehend whether and which TFs are responsible for the development of other lung cancer subtypes through this mechanism, and how we can exploit this knowledge for therapeutic treatment and prevention.
- Most studies so far have focused on describing hypermethylated promoters and downregulated target genes. However, the notable example of *TERT* upregulation through DNA hypermethylation requires further investigation of similar phenomena [106]. Although the detailed biological mechanisms for such activation remain unknown, hypermethylation might regulate the binding of methylation-sensitive TFs and/or the expression of nearby genes that ultimately influence expression. Alternatively, DNA methylation might result in the disruption of genome topology, driving aberrant regulatory interactions and abnormal expression of oncogenes in cancer [107,108]; thus it should be investigated in lung cancers. In addition, further studies integrating multi-omic data are required to elucidate the roles of global hypomethylation in lung and other cancer types. How concomitant hypermethylation and hypomethylation in CpG sites within the same gene regulates the gene expression of the target gene is another research question of interest.
- Although cell lines provide a useful model to study processes that can be observed in tumors, several studies have revealed that DNAm data from cell lines might not be representative of those from primary tumors. For instance, a global analysis indicated that cell lines are much more heavily methylated compared to primary tumors [198]. In concordance, Poirier et al. observed that DNA methylation profiles in primary SCLC are distinct from those of cell lines [126]. The source of the difference is unclear, but this means researchers should be cautious, and conclusions drawn from cell line models should be validated in primary tumors.
- Although different lung cancer subtypes (NSCLC vs. SCLC, LUAD vs. LUSC) have distinct genetic and molecular profiles, there have been limited direct comparisons of the DNAm epigenome between them. Future studies with a large sample size encompassing various lung cancer pathological entities would be required to systematically characterize the differences in their DNAm landscapes. The knowledge would be essential to understand the underlying mechanisms driving tumorigenesis of different subtypes, identify biomarkers for accurate differential diagnosis, and develop effective personalized treatments.
- While in lung cancers from smokers, tobacco-smoking carcinogens can provide a “fertile ground” for oncogenic mutations that drive tumor development [166] even in the early stages of lung carcinogenesis [199]. However, which exogenous/endogenous factors (e.g., environmental pollutants, inflammation, aging, chronic cellular stress) drive the epigenetic transformation of lung cancers in the absence of smoking carcinogens is unclear and warrants further investigation. With large datasets including genomic, epigenomic, and expression data from lung cancers in never-smokers, e.g., the Sherlock-*Lung* study [200], many of these questions could be answered.
- Recent studies have observed congruent genomic and DNAm evolutionary trajectories in lung cancer [82,201] as well as other cancers [202,203], highlighting the potential for epigenetic changes to provide a milieu for genomic changes driving tumorigenesis. Integrating the genomic and epigenomic profiles of lung cancer in future studies is essential to better comprehend lung tumor evolution.

Recent advances in single-cell DNA-methylation (sc-DNAm) profiling technologies have enabled the examination of cell-of-origins and cell type heterogeneity in cancers [204]. It is important to validate, at a single cell level, the correlation between DNAm and the expression of cancer driver genes in lung cancers, and to study the effect of methylation of different cell types in the tumor microenvironment on lung tumorigenesis. In addition, Hou et al. showed that copy number alterations proportionally affect mRNA expression, but not DNA methylation, at a single-cell level in liver cancer [205], suggesting that sc-DNAm has great potential for discriminating cell types. Future studies applying sc-DNAm in lung cancers could provide further insights into biological processes and evolutionary trajectories, and could identify markers for different subtypes and prognostic features.
